# Effect of Intermittent Fasting on Anthropometric Measurements, Metabolic Profile, and Hormones in Women with Polycystic Ovary Syndrome: A Systematic Review and Meta-Analysis

**DOI:** 10.3390/nu17152436

**Published:** 2025-07-25

**Authors:** Yazan Ranneh, Mohammed Hamsho, Wijdan Shkorfu, Merve Terzi, Abdulmannan Fadel

**Affiliations:** 1 Department of Nutrition and Dietetics, College of Pharmacy, Al-Ain University, Abu-Dhabi P.O. Box 64141, United Arab Emirates; 2 Department of Nutrition and Dietetics, Faculty of Health Science, Istanbul Yeni Yüzyıl University, Istanbul 34010, Turkey; hamsho2000001@hotmail.com (M.H.); merve.terzi@yeniyuzyil.edu.tr (M.T.); 3 Department of Nutrition and Dietetics, Faculty of Health Sciences, Bahçeşehir University, Istanbul 34349, Turkey; wijdanshkorfu@gmail.com; 4 Department of Nutrition and Health, College of Medicine and Health Sciences, United Arab Emirates University, Al Ain P.O. Box 1555, United Arab Emirates; afadel@uaeu.ac.ae

**Keywords:** polycystic ovary syndrome, PCOS, metabolic profile, hormonal profile, intermittent fasting, time-restricted eating, meta-analysis

## Abstract

Background: Polycystic Ovary Syndrome (PCOS) is a prevalent endocrine disorder characterized by excess body weight, hyperandrogenism, hyperglycemia, and insulin resistance often resulting in hirsutism and infertility. Dietary strategies have been shown to ameliorate metabolic disturbances, hormonal imbalances, and inflammation associated with PCOS. Recent evidence indicates that intermittent fasting (IF) could effectively enhance health outcomes and regulate circadian rhythm; however, its impact on PCOS remain unclear. Objective: Therefore, this systematic review and meta-analysis aims to examine the effect of IF on women diagnosed with PCOS. Methods: Comprehensive research was conducted across three major databases including PubMed, Scopus, and Web of Science without date restrictions. Meta-analysis was performed using Cochrane Review Manager Version 5.4 software. Results: Five studies fulfilled the inclusion criteria. IF significantly reduced body weight (MD = −4.25 kg, 95% CI: −7.71, −0.79; *p* = 0.02), BMI (MD = −2.05 kg/m^2^, 95% CI: −3.26, −0.85; *p* = 0.0008), fasting blood glucose (FBG; MD = −2.86 mg/dL, 95% CI: −4.83, −0.89; *p* = 0.004), fasting blood insulin (FBI; MD = −3.17 μU/mL, 95% CI: −5.18, −1.16; *p* = 0.002), insulin resistance (HOMA-IR; MD = −0.94, 95% CI: −1.39, −0.50; *p* < 0.0001), triglycerides (TG; MD = −40.71 mg/dL, 95% CI: −61.53, −19.90; *p* = 0.0001), dehydroepiandrosterone sulfate (DHEA-S; MD = −33.21 μg/dL, 95% CI: −57.29, −9.13; *p* = 0.007), free androgen index (FAI; MD = −1.61%, 95% CI: −2.76, −0.45; *p* = 0.006), and C-reactive protein (CRP; MD = −2.00 mg/L, 95% CI: −3.15, −0.85; *p* = 0.006), while increasing sex hormone-binding globulin (SHBG; SMD = 0.50, 95% CI: 0.22, 0.77; *p* = 0.004). No significant changes were observed in waist-to-hip ratio (WHR), total cholesterol (TC), LDL, HDL, total testosterone (TT), or anti-Mullerian hormone (AMH). Conclusions: IF represents a promising strategy for improving weight and metabolic, hormonal, and inflammatory profiles in women with PCOS. However, the existing evidence remains preliminary, necessitating further robust studies to substantiate these findings.

## 1. Introduction

The rising prevalence of chemical and endocrine-disrupting exposures, alongside interactions among lifestyle, environmental factors, and genetic predispositions, contributes significantly to increased susceptibility among women to gynecological disorders, notably polycystic ovary syndrome (PCOS) [1,2]. Affecting approximately 20% of women of reproductive age, PCOS manifests as a complex endocrinopathy characterized by morphological, hormonal, and metabolic abnormalities [1,3].

PCOS is frequently associated with metabolic dysfunctions evident in abnormal glycolipid profiles, predisposing affected individuals to insulin resistance, type 2 diabetes mellitus (T2DM), cardiovascular diseases (CVD), abdominal obesity, and certain malignancies [4,5,6]. Although extensive research exists, the precise etiology of PCOS remains elusive, with evidence supporting a multifactorial interplay involving genetics, hormones, and environmental influences [6,7].

Hormonal dysregulation in PCOS primarily involves hyperandrogenism, with elevated production of testosterone, androstenedione, and DHEA-S from ovarian theca cells and adrenal glands, contributing to hirsutism, acne, and anovulation. Disturbances in luteinizing hormone (LH) and follicle-stimulating hormone (FSH) further inhibit follicular maturation and ovulation, leading to ovarian cyst formation [8].

Furthermore, the coexistence of excess body weight (BW) and insulin resistance (IR) creates a reciprocal relationship that exacerbates the clinical presentation of PCOS [9,10]. Studies indicate that a substantial proportion of females diagnosed with PCOS, reaching up to 88%, fall either into overweight or obese categories [8]. It was also revealed that there is a significant correlation between the amount and distribution of abdominal adiposity in women with PCOS. Obese females with PCOS exhibited increased central abdominal fat and demonstrated markedly elevated insulin levels and diminished insulin sensitivity when compared to non-centrally obese control subjects [11,12]. Elevated insulin concentrations not only stimulate the ovarian synthesis of androgen but also suppress sex hormone-binding globulin (SHBG), resulting in increased levels of free testosterone [13].

Additionally, an imbalance in estrogen levels, characterized by elevated estrone (E1) relative to estradiol (E2) due to the peripheral conversion of androgens, results in chronic exposure to estrogen without the counterbalancing effects of progesterone [14]. This prolonged exposure may heighten the risk of developing endometrial hyperplasia, contributing to irregular menstrual cycles, infertility, and metabolic disturbances [14]. In addition, the association between obesity and chronic low-grade inflammation, further complicates the clinical picture, as this inflammatory state can intensify the symptoms [8,15,16].

Given the diverse array of signs and symptoms associated with PCOS, it is designated as a heterogeneous disorder leading to the diagnostic criteria that was established in 2003, the Rotterdam criteria, which is widely accepted as the primary diagnostic framework for PCOS, requiring the presence of at least two of the following three features: (1) hyperandrogenism, (2) oligo- or anovulation, and (3) polycystic ovaries [17].

Recent findings highlight the critical role of dietary modifications in alleviating the symptoms of PCOS and mitigating its related complications. These interventions are particularly effective in tackling issues such as inflammation, oxidative stress, anthropometry, and metabolic derangements [18,19,20]. Adopting this comprehensive strategy is vital for maximizing health outcomes for women suffering from PCOS.

Among various dietary strategies, intermittent fasting (IF) has emerged as a promising therapeutic intervention aimed at enhancing anthropometric measures and metabolic health, both of which are critical factors influencing the severity and progression of PCOS. A growing body of research indicates that IF and other forms of time-restricted eating (TRE) may positively affect IR, hyperglycemia, hyperlipidemia, inflammatory markers, and excess BW [21,22,23]. Furthermore, IF has been recognized for its capacity to influence the circadian clock, thereby initiating biochemical processes that regulate metabolism during fasting [24]. This relationship is particularly pertinent in the context of PCOS. Evidence suggests that disruptions in circadian rhythms can lead to symptoms resembling PCOS in animal models, including elevated testosterone levels and anovulation [25].

Although preliminary studies show promising results, the impact of these dietary patterns on women with PCOS remain ambiguous. Few systematic reviews evaluated the effectiveness of IF in modifying metabolic profile and fertility in PCOS. Kalsekar et al. (2024) conducted a study similar in concept to ours, albeit with minor methodological differences, which yielded mixed results [26]. Recently, Velissariou and his colleagues concluded that time-restricted feeding (TRF), a specific type of IF, is beneficial in alleviating fertility and reproductive hormones [27]. These results highlight the potential of IF to optimize health outcomes for women experiencing such an endocrine disorder.

However, due to lack of statistical analysis, the exact effect of IF on PCOS is still unknown. Therefore, in this systematic review we aim to quantitatively evaluate existing evidence that assess the impact of IF on metabolic profile, hormonal profile, and c-reactive protein (CRP) in PCOS women by applying statistical analysis methods such as meta-analysis

## 2. Methods

We performed a meta-analysis based on the guidelines of the Preferred Reporting Items for Systematic Reviews and Meta-Analyses (PRISMA) [28], and followed the methodological framework outlined in the Cochrane Handbook for Systematic Reviews of Intervention, version 6.3 [29]. The study protocol was registered in PROSPERO (CRD420251004903).

### 2.1. Search Strategy

A comprehensive literature search was conducted in three major electronic databases including PubMed, Web of Science, and Scopus up to the 5th of March 2025. The search targeted human interventional studies that assessed the effect of IF on PCOS. We used these key words in our search to search in title, abstract, and keywords (“polycystic ovary” OR “polycystic ovary syndrome” OR “polycystic ovarian syndrome” OR “pcos” OR “polycystic ovary disease” OR “polycystic ovarian disease”) AND (“intermittent fasting” OR “time restricted eating” OR “time restricted feeding” OR “if” OR “tre” OR “trf” OR “alternative day fasting” OR “adf”) AND (Intervention OR interventional OR “clinical trial” OR “randomized controlled trial” OR RCT). The search was independently conducted in duplicate by two authors (M.H. and W.S.) to ensure accuracy and reproducibility. In addition to database searching, the reference lists of all included articles were manually screened for additional eligible studies. Grey literature (such as theses, conference proceedings, and non-peer-reviewed reports) was not included in this review to ensure the inclusion of only peer-reviewed, high-quality publications. Discrepancies were resolved through discussion, and a third author (A.F.) was consulted when consensus was not achieved.

### 2.2. Inclusion Criteria

Studies were eligible for inclusion if they met the following criteria: (1) interventional studies (randomized or non-randomized) conducted in human adult female participants (≥18 years) diagnosed with PCOS according to Rotterdam criteria; (2) evaluated the impact of IF (including time-restricted eating or alternate-day fasting) on at least one of the following outcomes: anthropometric measures, metabolic parameters, hormonal markers, and inflammatory biomarkers; (3) reported pre- and post-intervention mean ± standard deviation (SD) values for eligible outcomes; and (4) published in peer-reviewed journals and written in English.

### 2.3. Exclusion Criteria

Studies were excluded if they met the following criteria: (1) they included participants with PCOS who were receiving concurrent pharmacological treatments (e.g., metformin, oral contraceptives) or surgical interventions (e.g., ovarian drilling) during the fasting period, which could confound the effects of intermittent fasting; (2) they combined intermittent fasting with other structured interventions such as exercise programs or hypocaloric diets not clearly distinguished from the IF protocol; (3) they lacked sufficient statistical data, such as pre- and post-intervention mean ± SD, or data not amenable to transformation for meta-analysis; (4) they did not clearly define the intermittent fasting protocol or lacked adherence monitoring; (5) they were duplicate publications or reported overlapping datasets, in which case the most recent or comprehensive version was included; or (6) they were non-peer-reviewed sources including conference abstracts, dissertations, and editorials, or were not published in English.

### 2.4. Data Extraction

After the selection process of articles, with regard to the inclusion and exclusion criteria, the following information (study, location, study type, duration, sample size, age, BMI, intervention, control, and method of caloric assessment) were extracted by two independent authors (W.S and M.H.) from the articles and listed in (Table 1).

### 2.5. Risk of Bias Assessment

All studies selected for retrieval were assessed by two independent reviewers (M.H. and Y.R.). The quality of the included randomized controlled trials was evaluated according to the Revised Cochrane risk-of-bias tool for randomized trials (RoB2) [30]. This tool has the following key parts: (1) random sequence generation, (2) allocation concealment, (3) blinding of participants and personnel, (4) blinding of outcome assessment, (5) incomplete outcome data, (6) selective reporting, and (7) other bias (other sources of bias that have been detected by the reviewer). Each item was categorized as having a low/unclear/high risk of bias. Accordingly, studies with more than two items of low risk were categorized as studies of good quality, studies with two items of low risk were considered studies of fair quality, and with fewer than two items with low risk of bias, they were considered studies of weak quality. Risk of Bias in Non-Randomized Studies-of Interventions (ROBINS-I) was applied to assess the risk of bias in non-randomized trials [31].

### 2.6. Statistical Analysis

The present meta-analysis was performed using the Cochrane Program Review Manager Version 5.4. Variables assessed in at least three interventional groups were included. In this regard, pre- and post-values of the mean ± SD of BW, body mass index (BMI), waist–hip ratio (WHR), fasting blood glucose (FBG), fasting blood insulin (FBI), Homeostasis Model Assessment for Insulin Resistance (HOMA-IR), total cholesterol (TC), high density lipoprotein (HDL), low density lipoprotein (LDL), triglyceride (TG), total testosterone (TT), SHBG, free androgen index (FAI), anti-Mullerian hormone (AMH), DHEA-s, and CRP were assessed. If the median with Q1 and Q3 was reported, the data were converted to mean and SD based on the method described here [32]. In order to apply mean difference in forest plots, units were converted into one measurement by using appropriate equations, when applicable. The random-effects model using the DerSimonian and Laird (DL) method was applied for pooling analysis to compensate for the heterogeneity of the studies [29]. Interstudy heterogeneity was explored quantitatively using Cochran’s Q and *I*^2^ statistics. *I*^2^ ≥ 50% indicates substantial heterogeneity, and *I*^2^ ≥ 75% indicates considerable heterogeneity [30]. *p*-values were considered statistically significant at <0.05.

## 3. Results

### 3.1. Literature Selection

**Figure 1:**
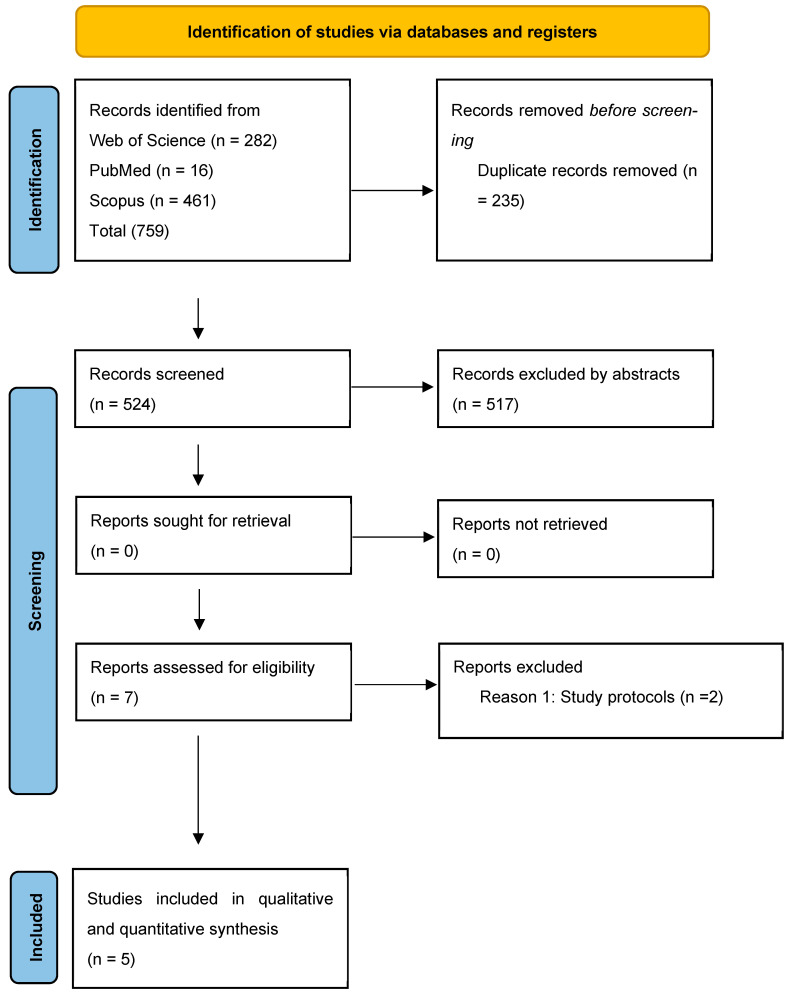
PRISMA flowchart of the included studies in this systematic review and meta-analysis.

A total of 759 citations were obtained from the initial search (Figure 1). All human interventional studies that investigated the impact of IF on PCOS women were included in this article. In total, 521 articles remained after excluding duplicates, 517 articles were excluded by the title or abstract, and 7 articles were eligible for inclusion in the systematic review and meta-analysis. Of the seven studies of interest, two were excluded for the same reason (study protocol) (Supplementary Materials). The remaining five studies were included in the qualitative and quantitative analysis. Characteristics of the included studies are provided in (Table 1).

**Table 1:** Characteristics of the included studies in the systematic review and meta-analysis.

Reference	Location	Study Type	Duration	Sample Size	Age	BMI	Intervention	Control	Method of Caloric Intake Assessment	Main Findings
[33] Li et al., 2021	China	Single arm interventional study	5 weeks	15	18–40	≥24 kg/m^2^	TRE 16:8, eat freely during prescribed period	N/A	Food diary using Boohee (version 7.4.2) software from the first to the last day.	↓ BW, ↓ BMI, ↓ BFM, ↓ BF %, ↓ VFA, ↓ FINS, ↓ HOMA-IR, ↓, ↓ TT, ↓ FAI, ↑ SHBG, ↓ IGF-1, ↓ ALT, ↓ hs-CRP, ↑ Menstrual cyclicity (73% resumed menses), ↔ TG/TC/LDL/HDL, ↔ MM
[34] Feyzioglu et al., 2023	Turkey	Single arm interventional study	6 weeks	30	<18 and >40 years	>30 or <18 kg/m^2^	TRE 16:8, eat freely during prescribed period	N/A	N/R	N/A
[35] Abu Salma et al., 2024	Jordan	RCT	6 months	Intervention (57) Control (29)	19–40 years	greater than 25 kg/m^2^	ADF (4:3), 18 fasting hours on non-consecutive days up to three times per week with 500 calorie restriction weekly.	Restriction of 500 calories/week	Food diary for two non-consecutive weekdays and one weekend day. The food records were analyzed using the Food Processor SQL Nutrition and Software, 2008 (ESHA Version 8.6)	BW, ↓ BMI, ↓ FM, ↓ BF %, ↓ VFA, ↓ WHR, ↑ ↑ MM, ↑ FFM
[36] Talebi et al., 2024	Iran	RCT	8 weeks	Intervention arm (A) (30), Intervention arm (B) (30), and control (30)	18–40 years	25–35 kg/m^2^	Arm (1): eTRE (14:10) + probiotic placebo + 25% calorie restriction. Arm (2): eTRE (14:10) + probiotic + 25% calorie restriction. Both groups were eating freely during prescribed period.	Calorie restriction by 25% + probiotic placebo	A 24-h food recall for 3 days at baseline, 2, 4, 6 and 8 weeks into the intervention. Dietary data were computed using Nutritionist IV software version 4.1 (First Databank, San Bruno, CA, USA) modified for Iranian foods.	↓ LDL-C/HDL-C/TC/ ↓ BW, ↓ hirsutism, ↓ acne score, ↓ HOMA-IR, ↓ FBG, ↓ SHBG, ↔ TT, ↔ AMH
[37] Talebi et al., 2025	Iran	RCT	8 weeks	Intervention arm (1) (30), Intervention arm (2) (30), and control (30)	18–40 years	25–35 kg/m^2^	Arm (1): eTRE (14:10) + probiotic placebo + 25% calorie restriction. Arm (2): eTRE (14:10) + probiotic + 25% calorie restriction. Both groups were eating freely during prescribed period.	Calorie restriction by 25% + probiotic placebo	A 24-h food recall for 3 days at baseline, 2, 4, 6 and 8 weeks into the intervention. Dietary data were computed using Nutritionist IV software version 4.1 (First Databank, San Bruno, CA) modified for Iranian foods.	↓ BW, ↓ BMI, ↓ WC, ↓ SBP (in eTRE groups), ↓ CRP (in all groups), ↑ TAC (in eTRE + probiotic and DCR), ↔ DBP, ↔ TOS, ↔ OSI

RCT: Randomized controlled trial. eTRE: Early time restricted eating. BW: Body weight. BMI: Body mass index. BFM: Body-fat mass. BF %: Body-fat percentage. VFA: Visceral fat area. FINS (FBI): Fasting blood insulin. MM: Muscle Mass. HOMA-IR: Homeostasis Model Assessment of Insulin Resistance. TT: Total testosterone. FAI: Free androgen index. SHBG: Sex-hormone-binding globulin. IGF-1: Insulin-like growth factor 1. ALT: Alanine aminotransferase. hs-CRP: High-sensitivity C-reactive protein. TG: Triglycerides. TC: Total cholesterol. LDL: Low-density-lipoprotein cholesterol. HDL: High-density-lipoprotein cholesterol. TOS: Total Oxidant Status. OSI: Oxidative Stress Index. TAC: Total Antioxidant Capacity. SBP: Systolic Blood Pressure. DBP: Diastolic Blood Pressure. ↑: increase significantly, ↓: decrease significantly, ↔: no significant change.

### 3.2. Studies’ Characteristics

A total of five studies were included in this systematic review and meta-analysis [33,34,35,36,37]. These studies included a total of (176) participants with an age range of 18–75. The type of fasting varied among the studies; the TRE diet was in four of them [33,34,36,37], and alternative day fasting (ADF) was in one of them [35]. The fasting period in TRE studies varied between 16:8 [33,34], and 14:10 [36,37]. However, fasting and eating windows differed among the included studies; fasting from 4 pm to 8 am [33], fasting from 9 pm to 1 pm [34], and fasting from 6 pm to 8 am (eTRE) [36,37]. One study included two interventional groups; one of them was TRE (arm A), and the other was TRE + probiotic supplementation (arm B) [36,37]. While some studies used food dairy [33,35], and 24 food recall [36,37] to assess the total caloric intake of participants, one of the included studies did not measure caloric intake [34]. Studies were conducted in various locations, two studies in Iran [36,37], one study in Turkey [34], one study in China [33], and one study in Jordan [35]. The duration of trial interventions ranged from 5 weeks to 8 months. All the studies included participants who were overweight and obese. BW was assessed in three studies [33,35,36], BMI was assessed in four studies [33,34,35,36], fat mass (FM) (kg), fat (%), and visceral fat were assessed in two studies [33,35], fat-free mass (FFM) and muscle mass were assessed in one study [35], skeletal muscle mass was assessed in one study [33], WHR was assessed in four studies [33,34,35,36], FBG, FBI, HOMA-IR and TG were assessed in three studies [33,34,36], TC was assessed in two studies [33,36], LDL was assessed in three studies [33,34,36], HDL was assessed in two studies [34,36], TT, SHBG, and FAI were assessed in three studies [33,34,36], AMH and DHEA-s were assessed in two studies [34,36], LH and FSH were assessed in two studies [33,34], E2, prolactin, and thyroid-stimulating hormone (TSH) were assessed in one study [34], and CRP was assessed in two studies [33,36].

### 3.3. Risk of Bias Assessment:

Risk-of-bias assessments were performed for all included studies. Among the three RCTs, two were judged to be at high risk of bias due to concerns in allocation concealment and outcome reporting, while one was rated as having some concerns. In accordance with RoB2, any study with a high-risk domain was classified as overall high risk. The two non-randomized studies were assessed using ROBINS-I and were rated as having a critical risk of bias due to lack of control groups and potential confounding. A summary of the risk-of-bias assessments is provided in Supplementary Figures S1–S3.

### 3.4. Effect of IF on Metabolic Profile

**Figure 2:**
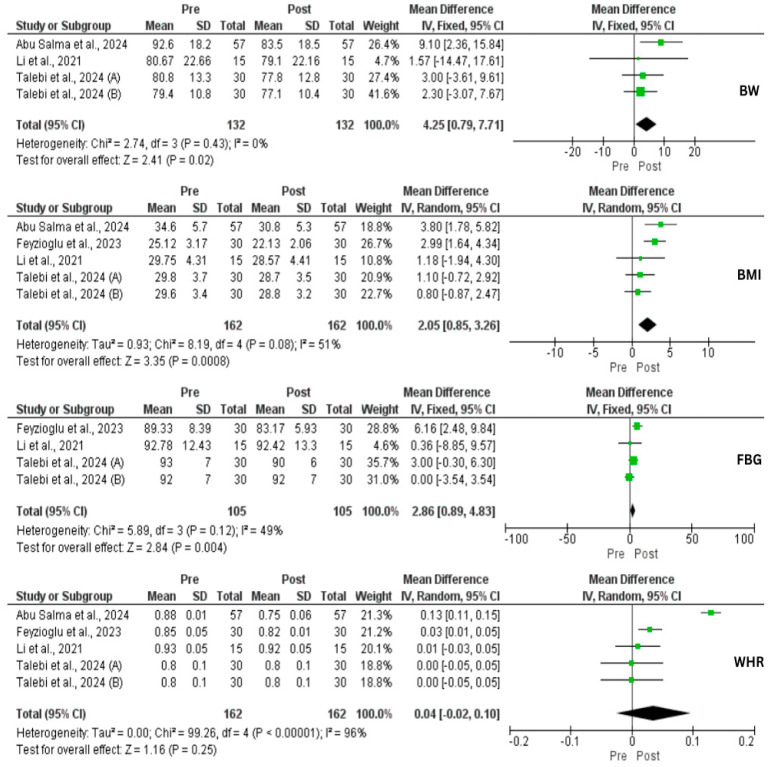
Forest plots for the body weight (BW), body mass index (BMI), fasting blood glucose (FBG), and waist–hip ratio (WHR) of intermittent fasting (IF) vs. non-intervention diet among polycystic ovarian syndrome (PCOS) patients [33,34,35,36,37].

**Figure 3:**
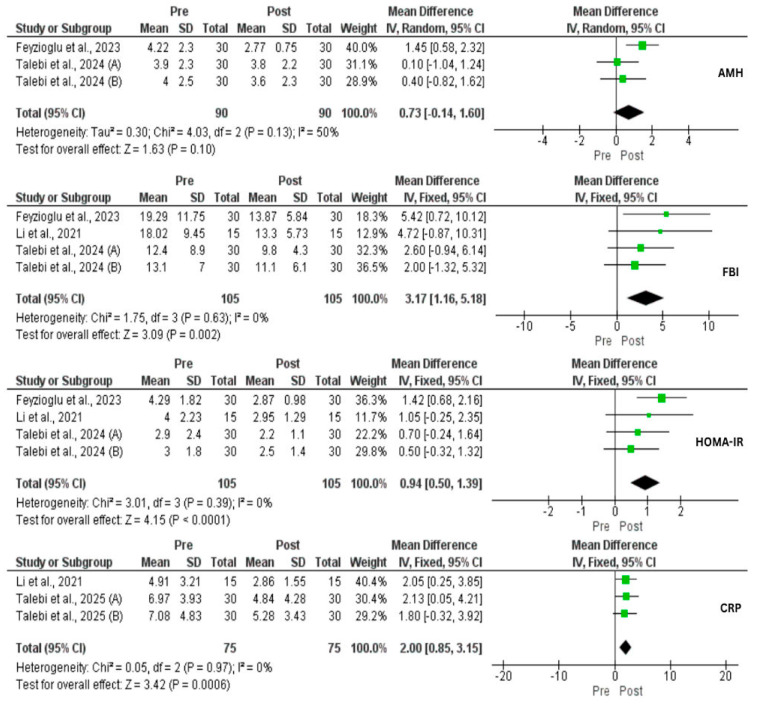
Forest plots for the Anti-Mullerian Hormone (AMH), fasting blood insulin (FBI), Homeostasis Model Assessment of Insulin Resistance (HOMA-IR), and C-Reactive Protein (CRP) of intermittent fasting vs. non-intervention diet among polycystic ovarian syndrome (PCOS) patients [33,34,36,37].

**Figure 4:**
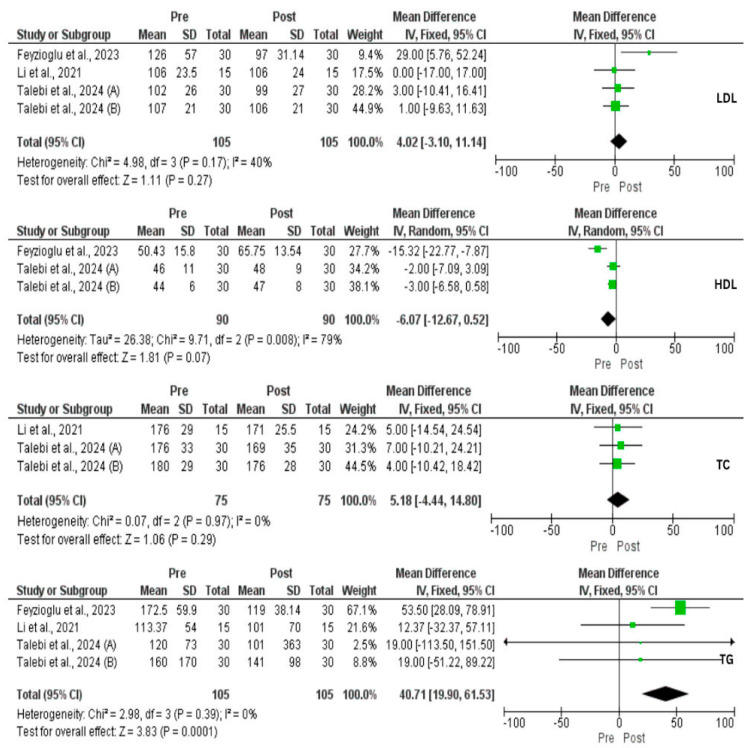
Forest plot for the low-density lipoproteins (LDL), high-density lipoproteins (HDL), total cholesterol (TC), and triglyceride (TG) of intermittent fasting vs. non-intervention diet among polycystic ovarian syndrome (PCOS) patients [33,34,36,37].

A total of three studies (four interventional groups) assessed the impact of IF on weight changes. There was a significant reduction in weight loss (MD = 4.25 kg, 95% CI: 0.79, 7.71, *p* = 0.02, *I*^2^ = 0%) and BMI (MD = 2.05, 95% CI: 0.85, 3.26, *p* = 0.0008, *I*^2^ = 51%) (Figure 2). In addition, a significant reduction in body FM (kg) (*p* = 0.01), body fat (%) (*p* = 0.01), and visceral fat (*p* = 0.01) was achieved [33,35]. In contrast, there was a significant increase in FFM (*p* = 0.01), and body muscle (*p* = 0.001) [35]. However, Li et al. [33] found no effect of IF on skeletal muscle mass (*p* = 0.06). Despite these improvements, our analysis did not find a reduction in WHR (*p* = 0.25). Moreover, IF exerted a significant decrease in FBG (MD = 2.86 mg/dL, 95% CI: 0.89, 4.83, *p* = 0.004, *I*^2^ = 49%), FBI (MD = 3.17 μU/mL, 95% CI: 1.16, 5.18, *p* = 0.002, *I*^2^ = 0%), HOMA-IR (MD = 0.94, 95% CI: 0.50, 1.39, *p* < 0.0001, *I*^2^ = 0%), and TG (MD = 40.71 mg/dL, 95% CI: 19.90, 61.53, *p* = 0.0001, *I*^2^ = 0%). On the other hand, IF did not affect TC, LDL, and HDL (Figure 2, Figure 3 and Figure 4).

### 3.5. Effect of IF on Hormones and CRP

**Figure 5:**
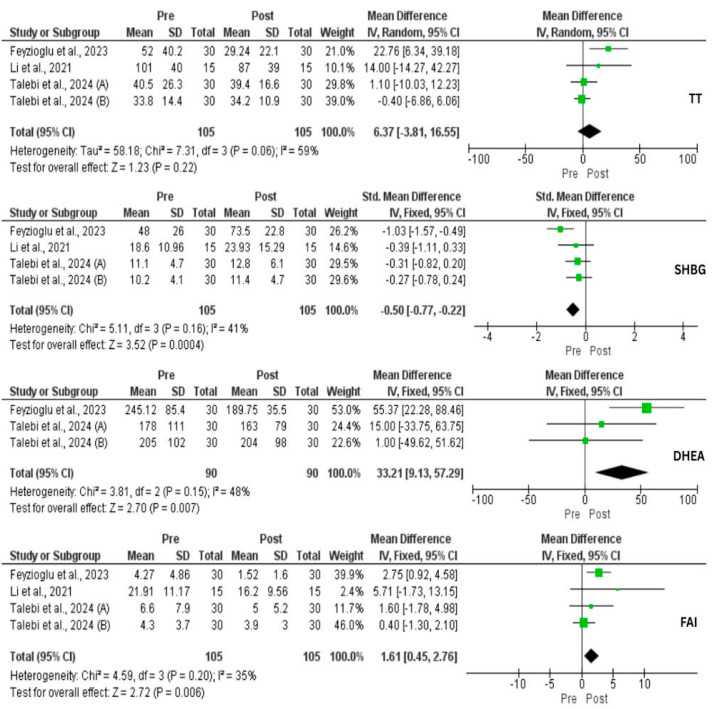
Forest plot for the total testosterone (TT), sex hormone binding globulin (SHBG), dehydroepiandrosterone (DHEA), and free androgen index (FAI) of intermittent fasting vs. non-intervention diet among polycystic ovarian syndrome (PCOS) patients [33,34,36,37].

Three studies assessed the effect of IF on TT, and no effect was observed (*p* = 0.22). However, DHEA-s was significantly decreased (MD = 33.21 µg/dL, 95% CI: 9.13, 57.29, *p* = 0.007, *I*^2^ = 48%). SHBG was significantly lower before IF compared to after IF (SMD = 0.50, 95% CI: −0.77, −0.22, *p* = 0.004, *I*^2^ = 41%). Subsequently, FAI was significantly reduced (MD = 1.61%, 95% CI: 0.45, 2.76, *p* = 0.006, *I*^2^ = 35%) (Figure 5). No statistical significance on AMH was found (*p* = 0.10) (Figure 3). Regarding LH and FSH, contradictory results were found in which Feyzioglu et al. [34] reported significant reductions while Li and his colleagues [33] did not find any effect. Additionally, there was a significant reduction in E2 and prolactin, and a significant increase in TSH [34]. Three studies investigated the impact of IF on CRP comprising 75 patients. As a result, there was a significant reduction at the end of the study (MD = 2 mg/L, 95% CI: 0.85, 3.15, *p* = 0.006, *I*^2^ = 0) (Figure 3).

## 4. Discussion

**PCOS** is a complex metabolic disorder characterized by hormonal disturbance, excess BW, IR, and elevated blood glucose and lipids. Development of PCOS can lead to T2DM and CVD which underscores the importance of early treatment [38,39]. Several dietary interventions have been proposed to alleviate PCOS-associated clinical symptoms and biomarkers. Among them, weight loss strategies have gained the highest attention [20,40]. IF is thought to effectively enhance health outcomes in patients with chronic diseases. Recently, several trials have investigated the effect of IF on PCOS. To our knowledge, this is the first systematic review and meta-analysis to investigate the impact of IF on women with PCOS. Our results indicate a significant reduction in BW, BMI, FM, VFM, FBG, FBI, HOMA-IR, TG, DHEA-s, E2, prolactin, TSH, FAI, and CRP, and an increase in FFM, and SHBG. Also, no effect on WHR, TC, HDL, LDL, and TT was observed. These results partially contrast with previously published systematic reviews, mainly due to lack of enough interventional studies and statistical analysis [26,27,41].

It is known that obesity causes several cardiometabolic dysfunctions including IR, elevated glucolipid profile, and hormonal disturbances [42]. In addition, obesity and overweight are the most common (88%) characteristics among PCOS patients in which a bidirectional relationship was reported in the literature [8]. Several studies found improvements in PCOS patients’ metabolic profile, hormones, and inflammation status following weight loss and dietary strategies. For instance, weight loss reduction significantly reduced HOMA-IR, IR, menstrual cycles, and FAI, but not sex hormones [19,43]. Although the relationship between diet and hormonal changes in PCOS is poorly studied, a recent meta-analysis reported a significant reduction in TT, and increased SHBG and FSH following a low carbohydrate diet [44]. Diet-induced weight loss caused a significant reduction in LH and an increase in SHBG. In our study, LH showed contradictory results. However, even when LH was not reduced, the menstrual cycle was normalized in 70% of patients [33]. Our analysis shows a statistically significant reduction in CRP levels by 2 mg/L. In PCOS rats, a reduction in CRP resulted in decreased leptin resistance and weight gain, increased energy expenditure, and improved insulin sensitivity [33]. Aligning with our result, weight loss caused a reduction in inflammatory biomarkers including CRP, IL-6, and TNF-α in PCOS women [45]. A network meta-analysis investigated the effect of various pharmacological and lifestyle modification interventions on PCOS parameters. A low calorie-diet has exerted the most effective treatment in reducing BMI. A low-calorie diet with metformin ranked highest in BW reduction. Therefore, dietary interventions, particularly hypocaloric diets, have been suggested as a first line treatment for PCOS [40].

In this context, IF approaches (e.g., TRE, ADF, and 5:2) are widely adopted to reduce BW and improve overall health. In the last decade, several RCTs and meta-analyses have investigated the impact of IF subtypes on various populations and health conditions. While few studies did not find benefit of IF, most studies reported significant weight loss and improvements in various biomarkers including glucolipid profile [46,47,48]. It is thought that IF benefits are not limited to weight loss but may also increase TEE and fecal energy excretion, and regulate circadian rhythm and hormones secretion [49,50]. On the other hand, recent evidence that compares IF and calorie restriction in an isocaloric state suggests that these improvements are solely explained by weight loss rather than IF [51]. This evidence is supported by one of the included studies; Talebi and his colleagues [36] assigned participants to three groups, TRE, TRE + probiotic, and control (calorie restriction). At the end of the study, there was no major difference between the groups. Despite this, the IF + probiotic group had a significant decrease in homa-b (*p* = 0.009) indicating a potentially positive impact of probiotic combined with IF, although all the groups had similar results at the end of the study. In addition, there was a significant reduction in PCOS symptoms, including hirsutism and acne, in all the groups [36].

In contrast, Abu Salma and his colleagues [35] demonstrated significant superiority of IF over CR on anthropometric measurements in PCOS. For instance, the IF group lost 9.2 kg while the CR group lost 2.4 kg during the study. A similar trend was observed in BMI and FM. Notably, sufficient information regarding the intervention, such as caloric intake during non-fasting days, was not provided. In addition, significant variations in baseline values were demonstrated, reflecting an inappropriate randomization method and potential selection bias, both of which are common biases in IF research as was mentioned previously [52]. Briefly, IF intervention has other dimensions that might not co-exist with other interventions such as religion beliefs, which can also indicate bias by investigators (e.g., show positive results to support their religion) and social media influencers leading to overestimation of results. Despite this, even under isocaloric state, IF produced a significant reduction in HOMA-IR (*p* = 0.02) and a considerable reduction in FBI (*p* = 0.08) which are key factors of PCOS progression [53].

Combining our study findings with existing evidence, IF intervention limits the time of food intake during the day, and thus total caloric intake, resulting in weight loss. Moderate weight loss (5–10%) has been shown to significantly improve insulin sensitivity and beta cell function which, in turn, decreases IR and FBG [54,55]. Interestingly, in PCOS women, even a 2% weight loss resulted in significant improvements in menstrual irregularities [55]. It was demonstrated that elevated insulin secretion activates LH receptors which stimulates testosterone secretion [56]. Subsequently, high LH levels are responsible for testosterone synthesis signaling in the ovarian theca cells. It was found that elevated LH levels increase free testosterone by reducing serum SHBG, thus amplifying hyperandrogenism and menstrual irregularity [51]. Therefore, providing effective weight loss strategies (e.g., diet and supplements) in PCOS patients is considered the first line treatment as it causes a series of events mediating the improvement in metabolic profile, hormonal profile, and inflammation [40,57]. Moreover, IF, specifically TRE, may provide additional health outcomes in PCOS women due to its regulatory mechanisms on circadian rhythm which have been shown to play a critical role in optimizing endocrinal functions [49]. Finally, these metabolic and hormonal regulations followed by weight loss may lead to improvements in PCOS main symptoms including hirsutism, acne, menstrual cycles, and fertility.

## 5. Strengths and Limitations

The current systematic review and meta-analysis provides novel comprehensive results of IF effects in PCOS women. Our findings are supported with statistical analyses which increases the robustness of our review. In addition, this article provides potential mechanism of action of IF in PCOS women and highlights the importance and directions of future studies. Heterogeneity among the studies in meta-analysis was low, indicating consistency in results among the studies. Despite this, several limitations must be considered in this article. The number of included studies (*n* = 5) and participants (*n* = 176) were very low, which limits the generalizability. In addition, baseline values varied among the studies which may contribute to inconsistent results, especially in sex hormones. Moreover, most included studies did not prescribe or follow the participants’ caloric intake, which makes it difficult to separate the true effect of IF intervention. Although Li et al. [33] instructed the participants to not change their habitual diet and to record their calorie intake during the experiment, aiming to reduce the bias arising from confounding factors such as calorie restriction rather than IF, the data of caloric intake were not reported. Meta-analysis was conducted as pre–post analysis which has less quality compared to intervention vs. control analysis. Also, although a risk-of-bias assessment was conducted, variability in study quality remains a concern and may influence the overall effect estimates. Due to the small number of studies included per outcome, we did not conduct formal assessments of publication bias such as funnel plots or Egger’s test. In addition, the heterogeneity in intermittent fasting protocols, intervention durations, and population characteristics precluded us from performing subgroup analyses or meta-regressions, which may have provided deeper insight into effect modifiers. Finally, the studies included provide data for short-term IF effects; long-term effects remain unexamined.

## 6. Conclusions

Future research should focus on conducting longitudinal trials examining the impact of IF on metabolic profile, hormonal profile, and inflammation. Additionally, study investigators should consider previously highlighted methodological limitations and solutions of current IF studies [52]. In conclusion, IF may be an effective approach for weight loss and achieving health benefits such as improvement in metabolic profile, hormonal profile, and inflammation in women diagnosed with PCOS. However, the evidence is still weak, and future studies are needed to confirm or deny our findings.

## Supplementary Materials

The following supporting information can be downloaded at: https://www.mdpi.com/article/10.3390/nu17152436/s1, Figure S1: Risk of bias summary of included RCTs; Figure S2: Risk of bias graph of included RCTs; Figure S3: Risk of bias summary of included non RCTs; PRISMA Checklist. References [58,59] are cited in the supplementary materials.
